# Omega-3 Fatty Acid Intake of Pregnant Women and Women of Childbearing Age in the United States: Potential for Deficiency?

**DOI:** 10.3390/nu9030197

**Published:** 2017-02-26

**Authors:** Tara M. Nordgren, Elizabeth Lyden, Ann Anderson-Berry, Corrine Hanson

**Affiliations:** 1 Pulmonary, Critical Care, Sleep & Allergy Division, Department of Internal Medicine, University of Nebraska Medical Center, 985910 Nebraska Medicine, Omaha, NE 68198-5910, USA; 2 Biostatistics Department, College of Public Health, University of Nebraska Medical Center, 984375 Nebraska Medical Center, Omaha, NE 68198-4375, USA; elyden@unmc.edu; 3 Department of Pediatrics, University of Nebraska Medical Center, 981205 Nebraska Medical Center, Omaha, NE 68198-1205, USA; alanders@unmc.edu; 4 Medical Nutrition Education Division, College of Allied Health Professions, University of Nebraska Medical Center, 984045 Nebraska Medical Center, Omaha, NE 68198-4045, USA; ckhanson@unmc.edu

**Keywords:** omega-3 fatty acid, women, childbearing, diet, pregnancy, socioeconomic

## Abstract

Omega-3 fatty acids play critical roles during fetal growth and development with increased intakes associated with improved maternal-fetal outcomes. Omega-3 fatty acid intake in Western diets is low, and the impact of socioeconomic factors on omega-3 fatty acid intake in pregnant women and women of childbearing age has not been reported. We used the National Health and Nutrition Examination Survey (NHANES) cycles 2003–2012 to assess the relationship between omega-3 fatty acid intake and socioeconomic factors in women of childbearing age. Out of 7266 eligible participants, 6478 were women of childbearing age, while 788 were identified as pregnant at the time of the survey. Mean EPA+DHA intake of the population was 89.0 mg with no significant difference between pregnant and non-pregnant women. By univariate and multivariate analyses adjusting for confounders, omega-3 fatty acid intake was significantly associated with poverty-to-income ratio, race, and educational attainment. Our results demonstrate that omega-3 fatty acid intake is a concern in pregnant women and women of childbearing age in the United States, and that socioeconomically disadvantaged populations are more susceptible to potential deficiencies. Strategies to increase omega-3 fatty acid intake in these populations could have the potential to improve maternal and infant health outcomes.

## 1. Introduction

Maternal diet is critical for a successful pregnancy, as well as fetal health outcomes [1,2,3]. The hypothesis that early life diet in utero increases the vulnerability of the offspring to the development of poor outcomes and disease is now well accepted [4,5]. Several studies have established that quantity and quality of dietary fats consumed during pregnancy have profound health implications during and after pregnancy [6,7]. Omega-3 fatty acids play critical roles during fetal growth and development, and higher intakes of omega-3 fatty acids during pregnancy have been associated with decreased maternal depression [6], reduced rates of intrauterine growth restriction [6], preterm birth [8,9,10], reduced allergies and asthma in children [11], and improved neurocognitive outcomes in the offspring [6].

Despite the importance of these fatty acids in maternal-fetal health, omega-3 fatty acid intake is typically very low in the Western diet [12,13]. Socioeconomic factors have been shown to impact the overall quality of diet, such as intakes of fruits and vegetables [14,15,16,17,18], however the impact of socioeconomic indicators on intake of omega-3 fatty acids is unknown. The National Health and Nutrition Examination Survey (NHANES) contains dietary intake of omega-3 fatty acids, including DHA and EPA, and measures of socioeconomic status, allowing us to evaluate these relationships. Therefore, the objective of this study was to use NHANES data to examine omega-3 fatty acid intake of women of childbearing age in the United States, and to assess the impact of poverty, race, food security, and other socioeconomic factors on omega-3 fatty acid status.

## 2. Materials and Methods

### 2.1. Subjects

Women who were 14–45 years of age and women who were identified as pregnant from NHANES cycles 2003–2012 were included in the analysis (detailed information regarding the collection and analyses of the NHANES datasets are available on the Centers for Disease Control and Prevention website, accessible at http://wwwn.cdc.gov/Nchs/Nhanes/ [19]). Waves earlier than 2003 were not included due to large amounts of missing data (up to 85%) on socioeconomic indices. Participants with energy intake greater than or less than the plausible intake (<600 or ˃6000 kcal/day) were excluded from the analysis, as were women missing information on pregnancy status. The final number of eligible participants was 7266.

### 2.2. Dietary Assessment

The main outcome variable was the dietary intake of omega-3 fatty acids DHA and EPA. The average EPA and DHA intakes for each participant were used to calculate a sum value of the two estimates (EPA+DHA). In other studies, average EPA, DHA, and their summation have been shown to be highly correlated [20,21]. However, since DHA is specifically recommended during pregnancy [22,23], we also evaluated DHA and EPA separately.

Dietary intake in the NHANES survey was determined from two interviewer-administered 24-h recalls using methodology developed and validated by the U.S. Department of Agriculture. The dietary recalls were conducted in English or Spanish in study participants who were 12 years and older. Three to 10 days later, all participants were asked to complete a second 24-h dietary recall interview by telephone.

Use of dietary supplements or prenatal vitamins containing omega-3 fatty acids was evaluated through the NHANES supplement files. Supplements containing the ingredient codes matching “alpha-linolenic acid (ALA), omega-3, docosahexaenoic acid (DHA) or eicosapentaenoic acid (EPA)” from the Dietary Supplement Ingredient Database (Release 3) were identified. The identified omega-3 supplements were then matched to the individual’s file to create a dichotomous (Y/N) variable for supplement intake in the last 30 days.

### 2.3. Other Covariates

Poverty-to-income ratio (PIR) was used as an index of socioeconomic status. The PIR is the ratio of household income to the poverty threshold after accounting for inflation and family size [24,25]. The PIR is used to determine eligibility for means-tested government-sponsored assistance programs relevant for women, particularly the Special Supplemental Nutrition Program for Women, Infants, and Children (WIC) [14].

Race/ethnicity was categorized as Hispanic, non-Hispanic white, non-Hispanic Black, and Other (including multi-racial). Education level was categorized as less than high school, high school diploma recipient, or GED (General Educational Development test), and greater than high school education.

Food insecurity was measured with the 18-item US Food Security Survey Module [26]. Questions are ordered by severity and attribute-related experiences or behaviors to insufficient resources to buy food over the past 12 months. A raw score was created by summing the affirmative responses of the 18 questions, with a higher score reflecting higher concentrations of food insecurity. Categories were then assigned on the basis of guidelines from the USDA: 0, full food security; 1–2, marginal food security; 3–5 (households without children), or 3–7 (households with children), low food security; and 6–10 (households without children) or 8–18 (households with children), very low food security. Food insecurity refers to households reporting low or very low food security, and we dichotomized food security into yes/no categories. Supplemental Nutrition Assistance Program (SNAP) participation was assessed with the question, “in the last 12 months, did you or any members of your household receive Food Stamp benefits?” Both food insecurity and SNAP participation are measured at the household level.

### 2.4. Statistical Analysis

Descriptive statistics (counts and percentages and means and standard deviations) are shown for all participants. SAS version 9.4 (SAS, Cary, NC, USA) was used for all statistical analyses. Survey procedures in this software package incorporate sample weights and adjust analyses for the complex sample design of the survey. Survey sample weights were used in all analyses to determine estimates that were representative of the U.S. civilian, non-institutionalized population. The SAS procedures PROC SURVEYFREQ, PROC SURVEYMEANS, PROC SURVEYLOGISTIC, and PROC SURVEYREG were used in computing descriptive analysis and doing regression analysis, because these procedures accounted for the weighted data and complex design of the sample. The results of the descriptive analysis for categorical variables are represented by counts, percentages and weighted frequencies. Means, standard errors and 95% confidence intervals were used for continuous variables. Associations between categorical variables were determined using the Wald chi-square test that accounts for the complex survey design. The *p*-values for the comparisons of continuous data between groups (e.g., omega-3 fatty acid categories and PIR groups) were obtained from “PROC SURVEYREG”, a SAS procedure that performs regression analysis for sample survey data. Potential confounding variables were chosen based on prior associations in the literature and significance in the univariate models. The final multivariate model included age, energy intake, race/ethnicity, PIR, NHANES wave, education level, and pregnancy status. A *p*-value of < 0.05 was considered statistically significant.

## 3. Results

The final number of eligible participants was 7266. Of these, 6478 were women of childbearing age, while 788 were identified as pregnant at the time of the survey. The demographic characteristics of the sample by pregnancy status are given in Table 1.

There were no differences in DHA, EPA, or DHA+EPA intake between the pregnant and the non-pregnant women (*p* = 0.79, 0.71, and 0.75 for DHA, EPA, and DHA+EPA respectively), therefore these populations were combined for analysis of relationships with socioeconomic factors. In the univariate analysis, a statistically significant association was seen between omega-3 fatty acid intake and PIR (*p* = 0.03, *p* = 0.03, and 0.03 for EPA, DHA, and EPA+DHA, respectively). Omega-3 fatty acid intake also differed significantly by NHANES wave (Table 2), race (Table 3), and educational attainment (Table 4).

There was no association between omega-3 fatty acid intake and food security (*p* = 0.17, 0.12, and 0.14 for EPA, DHA, and EPA+DHA, respectively) or SNAP use (*p* = 0.71, 0.58, and 0.62 for EPA, DHA, and EPA+DHA, respectively).

After adjusting for relevant confounders, including age, BMI, energy intake, pregnancy status and NHANES wave, significant associations were maintained between PIR, race and education level and intake of DHA, EPA, and DHA+EPA. The significant results of the multivariate regression models are shown in Table 5.

When the proportion of women who use a dietary supplement containing omega-3 fatty acids was evaluated, 5.7% of all participants took an omega-3 containing supplement, while 94.3% of participants did not. Omega-3 fatty acid-containing supplement usage was significantly associated with pregnancy status, poverty-to-income ratio, educational attainment, and race, as shown in Table 6.

## 4. Discussion

This study is the first to examine a wide range of socioeconomic indicators on the intake of omega-3 fatty acids in a nationally representative sample of pregnant women and women of childbearing age. Our study shows that lower poverty status, race, and lower educational attainment increase the risk of suboptimal intake of these essential compounds.

Daily intake recommendations for omega-3 fatty acids vary widely, and recommended daily allowance or dietary reference intakes have not been set for DHA or EPA. The exclusion of DHA and EPA from the dietary reference intakes issues by the Institute of Medicine of the National Academies is largely due to the limited evidence for quantity-based effects of the fatty acids that was available in the 1990s and early 2000s [12]. However, many studies performed in more recent years have demonstrated the healthful benefits of these fatty acids, and numerous groups have issued recommendations for omega-3 fatty acid intakes based on age, health, and other factors [12,23]. The members of the Workshop on the Essentiality of and Recommended Dietary Intakes for Omega-6 and Omega-3 Fatty Acid in 1999 recommended an adequate intake level of DHA+EPA in adults to be at least 650 mg/day, including at least 220 mg/day each of DHA and EPA, with DHA intake in pregnant and lactating women increased to at least 300 mg/day [23]. Other organizations have given similar recommendations, with the World Health Organization suggesting 200–500 mg/day of EPA+DHA [27], and numerous organizations encouraging two servings of fatty fish per week to reach an approximately 450–500 mg/day allowance [12]. Variation in these recommended levels are likely due to the wide range of dosing that have been used in clinical studies investigating the beneficial actions of these fatty acids, although the recommendations do indicate a general consensus suggesting a minimum requirement of 200 mg/day of EPA+DHA. Despite these recommendations, a study of the 1999–2000 NHANES data indicated average intake of 100 mg/day in the United States population [13]. Our findings indicate that in women of childbearing age, average intake of DHA+EPA is 89 mg/day. Mean DHA+EPA intake of men between the same age range of 14–45 years was 119 mg/day, significantly increased compared to women (*p* < 0.001). While we did find statistically significant differences in DHA and EPA intake based on NHANES wave (Table 2), we are not certain that these differences are of clinical significance, giving their low overall values compared with the discussed recommended values. Furthermore, intake of DHA, recommended to be at least 300 mg/day in pregnant women, was not significantly different between pregnant and non-pregnant women, and was only 66 mg/day in pregnant women and 58 mg/day in non-pregnant women of childbearing age. While these intake values did not include supplementation, our findings from these NHANES surveys indicate only 1.8% of non-pregnant and 9.0% of pregnant women took supplements containing EPA and/or DHA.

Furthermore, our data indicate that socioeconomically disadvantaged populations are particularly at risk for even lower levels of omega-3 intake. With increasing poverty, our data indicate decreased omega-3 fatty acid intake in women of childbearing age, regardless of pregnancy status (*p* = 0.02), and women with educational attainment beyond a high school diploma have an average EPA+DHA intake of 103 mg/day, while women with a high school diploma as highest degree or not achieving a high school diploma had average intakes of 66 mg/day and 83 mg/day, respectively (*p* = 0.0004). Race was also significantly associated with omega-3 fatty acid intake (*p* < 0.0001); non-Hispanic white women of childbearing age had the lowest intake of EPA+DHA, averaging 78 mg/day compared to Hispanic women (94 mg/day), non-Hispanic Black women (112 mg/day), and women of other races, including multi-racial (142 mg/day). These findings indicate numerous risk factors for insufficient omega-3 fatty acid intake in pregnant women and women of childbearing age when compared to suggested intake values [12,23].

Omega-3 supplementation in mothers and infants is associated with numerous positive health outcomes in mother and child [6,28]. Higher intake of omega-3 fatty acids in mothers is associated with reduced risk for depression and intrauterine growth restriction, increased infant birth weight, as well as reduced risk of preterm birth [6,7,8,9,10]. Numerous studies have found improved developmental and cognitive outcomes in infants and children with high or supplemented omega-3 fatty acid intake (including maternal intake), including increases in visual acuity and problem solving [6,29,30]. Although some endogenous synthesis of DHA from dietary fatty acid precursors EPA and ALA can occur, the primary source for fetal and infant DHA intake is dependent on maternal intake [28,31,32]. Specifically, maternal DHA stores are mobilized in the third trimester of pregnancy, and placental transfer of DHA is preferential to other fatty acids, including arachidonic acid and EPA [28]. Through the first two years of a child’s life, high levels of DHA are preferentially incorporated into the infant brain [28]. In this early timespan, much of an infant’s omega-3 intake is from maternal stores during pregnancy and in breastmilk or formula [33,34,35]. A breastfed infant’s red blood cell membrane DHA content, an indicator of intake, is associated with mother’s omega-3 fatty acid intake [35], corroborating the relationship between maternal and child omega-3 statuses.

The mechanisms underlying the beneficial effects of omega-3 fatty acids are not completely understood. Although, emerging research reveals the role of these lipids in the biosynthesis of bioactive signaling molecules [36]. Following uptake of long-chain polyunsaturated fatty acids into cell phospholipid membranes, signaling events activate the cleavage of these lipids from the membrane to serve as substrates for the biosynthesis of signaling molecules [37,38,39]. In this regard, the incorporation of these fatty acids into membranes provides pools of substrate for future cell responses, and membrane incorporation is based on dietary availability [38]. Of interest, while the omega-6 fatty acid arachidonic acid serves as the substrate for a number of pro-inflammatory lipid mediators, including prostaglandins and leukotrienes, EPA and DHA are substrates for the production of lipid mediators, including resolvins, protectins, and maresins, that are involved in the active regulation of inflammation resolution and repair processes [36,40,41,42]. These omega-3 fatty acid-derived pro-resolving mediators have been found in high levels in breastmilk (~100-fold increase in breastmilk compared to plasma [43]), suggesting a biological role for the mediators in infant health or development [43,44]. These findings imply the value of omega-3 intake in maternal and infant diets, although additional studies are needed to identify the association between omega-3 fatty acid intake, pro-resolving lipid mediator production, and the beneficial effects to mother and child.

Recent studies have raised concerns regarding nutrient intake in the United States, especially with nutrients that are of concern during pregnancy. Data from 2003–2008 NHANES cycles demonstrate that women of childbearing age in the United States are not meeting nutrient guidelines for several nutrients, with distinct differences present between ethnic groups and socioeconomic strata [14,45,46]. Lower income individuals consumed lower quality foods when compared to those with higher incomes as measured by the Alternate Healthy Eating Index, which includes omega-3 fatty acid intake as one of the components of a healthy diet [47]. Many pregnant women and women of childbearing age have also been shown to consume less than the recommended servings of seafood per week [48]. As reviewed by Makrides et al., studies consistently show improved infant birth weights in women taking omega-3 fatty acid supplements, regardless of income status [7]. Additional work is necessary to clarify the effect of socioeconomic status on omega-3 fatty acid-associated neurocognitive outcomes in infants, but data indicate improved neurobehavioral outcomes in preterm infants when mothers were supplemented while producing milk [7].

Multiple factors contribute to low-income individuals having high risk of poor quality diet, including lack of access to grocery stores and high cost of healthy foods [16]. Individuals in the lowest income households are the least likely to consume vegetables and fruits [15,17,18], and are more likely to consume foods high in fat and sugar and low in fiber [17,18]. This data raises significant concerns that access to foods with high nutrient density is not evenly distributed, and as a result, there are likely populations at risk for nutritional deficiencies.

Use of prenatal vitamins may not be a contributing factor toward closing the gap by socioeconomic group, as pre-conception use of multivitamins in the United States is often poor. Our findings indicate only 1.8% of non-pregnant women of childbearing age use omega-3 fatty acid-containing supplements. Furthermore, the inclusion of omega-3 fatty acids in prenatal vitamins is not standard, despite being identified as an important nutrient in pregnant women [22,23]. Furthermore, there is precedence for the need for sufficient omega-3 fatty acid intake both prior to and during pregnancy; numerous studies have investigated the bioavailability and incorporation kinetics of omega-3 fatty acids in serum, red blood cell lipid membranes, and various tissues [49,50,51,52,53]. Tissue uptake kinetics indicate EPA half-maximal levels in blood rise rapidly (3–5 days) and reach half-maximal red blood cell membrane incorporation in one month [49], but DHA incorporation kinetics are slower and more erratic [49]. In pregnant women, supplementation initiated during pregnancy with 100 mg/day DHA [54] or 185 mg/day DHA [55] did not impact cord blood omega-3 fatty acid composition; higher levels, ranging from 528–2700 grams/day of omega-3 or DHA supplementation during pregnancy have been shown to increase cord blood omega-3 fatty acid incorporation [55,56,57]. It has been posited that due to fat mobilization during the third trimester of pregnancy, supplementation with omega-3 fatty acids may not be necessary to ensure neonate sufficiency [58]. However, findings suggest omega-3 fatty acid incorporation into fat takes longer than red blood cell incorporation, with half-maximal uptakes of DHA and EPA by adipose tissue greater than one year during continuous daily supplementation [49]. Furthermore, maternal and neonatal PUFA statuses are correlated [59,60] , and typical dietary consumption patterns of omega-3 fatty acids by women of childbearing age significantly impacts maternal omega-3 fatty acid status during pregnancy and correlates with infant status at birth [60]. Although lower-dose omega-3 fatty acid supplementation during pregnancy may not alter cord blood omega-3 status, it could limit the typical decline of maternal DHA status during the third trimester of pregnancy [54]. This is of clear value to the mother; additionally, maternal DHA stores recover slowly following birth, and data indicate that successive pregnancies and infants reflect the maternal depleted status with maternal and infant omega-3 fatty acid levels significantly lower in subsequent pregnancies [59,61]. Together, these data support the importance of omega-3 fatty acid dietary intake in women of childbearing age, including prior to, during, and following pregnancy.

Our study has several limitations. The cross-sectional nature of NHANES makes it difficult to infer any causation, and it is also possible that confounding occurred from variables not considered in our analysis. Additionally, dietary intake in NHANES is based on two 24-h recalls, which may not be representative of usual intake, as day-to-day intake of specific food items like fish can be highly variable. However, a single 24-h recall is considered adequate for estimates of group means [62].

## 5. Conclusions

Our results demonstrate that omega-3 fatty acid intake in pregnant women and women of childbearing age may be a concern in socioeconomically disadvantaged populations of the United States. More research is needed to determine what type of interventions will be best for specific populations, especially the low-income populations that bear a greater burden of poor diet quality and adverse maternal-child outcomes. As the population of the United States becomes more diverse, issues regarding health and disparities in diet become even more salient.

## Figures and Tables

**Table 1:** Participant characteristics by pregnancy status.

Characteristic	Pregnant Women	Non-Pregnant Women	*p*-Value
	(*n* = 788; 10.8%)	(*n* = 6478; 89.2%)	
Continuous variables: Mean (SE ^1^)			
Age (years)	28.1 (0.33)	31.2 (0.18)	˂0.0001
Body Mass Index (kg/m^2^) ^2^	29.4 (0.37)	27.7 (0.15)	˂0.0001
Family PIR (0–18 score)	2.8 (0.11)	2.7 (0.04)	0.73
Energy Intake (kcal)	2144.0 (40.86)	2225.3 (8.72)	˂0.0001
DHA Intake (mg)	66.4 (0.006)	58.3 (0.002)	0.79
EPA Intake (mg)	34.4 (0.004)	30.2 (0.002)	0.71
DHA+EPA Intake (mg)	100.8 (0.010)	88.5 (0.004)	0.75
Categorical variables: *n* (%)			
Race			
Non-Hispanic White	317 (4.6)	2533 (95.4)	0.0005
Hispanic	268 (7.8)	1894 (92.2)	
Non-Hispanic Black	149 (7.3)	1592 (92.7)	
Other Race–Including Multi-Racial	54 (6.8)	459 (93.2)	
Education Level			
˂HS	185 (1.2)	1057 (14.8)	0.18
HS or equivalent	141 (1.2)	1027 (19.4)	
˂HS	363 (3.6)	2914 (60.0)	
SNAP Use			
Yes	163 (5.5)	1255 (76.2)	0.06
No	10 (0.60)	255 (17.6)	
Food Security Status			
Full	546 (4.4)	4212 (69.7)	0.32
Marginal	98 (0.58)	882 (9.7)	
Low	76 (0.44)	772 (8.5)	
Very Low	48 (0.28)	530 (6.4)	

^1^ SE: Standard Error; ^2^ BMI at time of enrollment in NHANES; values thus represent pregnancy BMI in individuals identifying as pregnant at the time of enrollment.

**Table 2:** Omega 3 fatty acid intake by NHANES wave ^1^.

Wave	Variable	*n*	Mean (SE ^2^), mg	95% CI for Mean
2003–2004	EPA	1820	27.8 (2.7)	22.2, 33.2
	DHA	1820	55.7 (4.0)	47.7, 63.8
	EPA+DHA	1820	83.5 (6.7)	70.0, 97.0
2005–2006	EPA	2022	40.0 (4.3)	31.4, 48.6
	DHA	2022	71.7 (6.3)	59.1, 84.2
	EPA+DHA	2022	111.7 (10.4)	91.0, 130.3
2007–2008	EPA	1098	26.7 (1.9)	23.3, 30.4
	DHA	1098	53.8 (3.2)	47.4, 60.1
	EPA+DHA	1098	80.6 (4.9)	70.7, 90.4
2009–2010	EPA	1299	35.0 (8.3)	18.5, 51.5
	DHA	1299	67.1 (11.8)	43.5, 90.1
	EPA+DHA	1299	102.1 (20.0)	62.2, 142.0
2011–2012	EPA	1027	20.5 (2.3)	16.0, 25.0
	DHA	1027	42.4 (3.3)	35.8, 48.9
	EPA+DHA	1027	62.9 (5.4)	52.2, 73.7

^1^ *p*-Value for EPA intake between waves = 0.0002, DHA and EPA+DHA both ˂0.0001; ^2^ SE: Standard Error.

**Table 3:** Omega-3 fatty acid intake by race ^1^.

Race/Ethnicity	Variable	*n*	Mean (SE ^2^), mg	95% CI for Mean
Hispanic	EPA	2162	31.0 (3.2)	24.6, 37.3
	DHA	2162	62.5 (3.9)	54.6, 70.4
	EPA+DHA	2162	93.5 (7.1)	79.4, 107.6
Non-Hispanic White	EPA	2850	26.6 (3.1)	20.4, 32.6
	DHA	2850	51.4 (4.1)	43.2, 59.5
	EPA+DHA	2850	78.0 (7.1)	63.8, 92.1
Non-Hispanic Black	EPA	1741	38.7 (3.5)	31.7, 45.6
	DHA	1741	73.0 (5.2)	62.6, 83.2
	EPA+DHA	1741	111.7 (8.6)	91.7, 12.8
Other Race—Including Multi-Racial	EPA	513	49.7 (4.6)	40.5, 58.9
	DHA	513	91.7 (8.1)	75.6, 107.9
	EPA+DHA	513	141.4 (12.5)	116.5, 166.5

^1^ *p* ≤ 0.0001 for EPA, DHA, and EPA+DHA; ^2^ SE: Standard Error.

**Table 4:** Omega-3 fatty acid intake and educational attainment ^1^.

Education	Variable	*n*	Mean (SE ^2^), mg	95% CI for Mean
<HS ^3^	EPA	1242	27.1 (2.9)	21.2, 33.0
	DHA	1242	56.2 (4.4)	47.4, 65.0
	EPA+DHA	1242	83.4 (7.2)	68.9, 97.8
HS/GED ^4^	EPA	1168	21.58 (2.2)	17.1, 26.0
	DHA	1168	44.0 (3.1)	37.7, 50.3
	EPA+DHA	1168	65.6 (5.2)	55.2, 76.0
>HS	EPA	3277	35.7 (3.3)	29.2, 42.3
	DHA	3277	66.9 (4.5)	57.8, 76.0
	EPA+DHA	3277	102.7 (7.8)	87.1, 118.3

^1^ *p* = 0.0025, 0.0001, and 0.0004 for EPA, DHA, and EPA+DHA, respectively. ^2^ SE: Standard Error; ^3^ HS: high school diploma; ^4^ GED: General Educational Development test.

**Table 5:** Results of multivariate regression models of socioeconomic indicators and omega-3 fatty acid intake ^1^.

Socioeconomic Indicator	EPA (mg)		DHA (mg)		EPA+DHA (mg)	
	β	*p*-Value	β	*p*-Value	β	*p*-Value
Poverty-Income Ratio (PIR)	3.1	0.03	4.5	0.03	7.6	0.03
Educational Attainment						
˂HS ^2^ vs. ˃HS	−6.4	0.09	−9.2	0.08	−15.5	0.08
HS/GED ^3^ vs. ˃HS	−11.5	0.0003	−19.6	<0.0001	−31.0	<0.0001
Race						
Non-Hispanic White vs. Black	17.8	<0.0001	30.4	<0.0001	48.2	<0.0001
Non-Hispanic White vs. Hispanic	11.8	0.01	23.4	0.0001	35.3	0.001
Non-Hispanic White vs. Other	24.0	0.0001	42.4	<0.0001	66.5	<0.0001

^1^ Models adjusted for age, BMI, energy intake, and NHANES wave. ^2^ HS: high school diploma; ^3^ GED: General Educational Development test.

**Table 6:** Omega-3 fatty acid-containing supplement usage.

Omega-3 Supplement Usage	*n*	Percent	*p* Value
Pregnancy Status			
Not Pregnant			
Yes	85	1.8	
No	6388	98.2	
Pregnant			
Yes	24	9.0	<0.0001
No	764	91.0	
PIR Ratio			
PIR ≤ 1.85			
Yes	33	1.2	
No	3484	98.8	
PIR > 1.85			
Yes	71	2.9	0.0003
No	3270	97.1	
Educational Attainment			
<HS ^1^			
Yes	7	0.9	
No	1234	99.1	
HS/GED ^2^			
Yes	16	1.5	
No	1151	98.5	
>HS			
Yes	82	3.0	0.0048
No	3193	97.0	
Race/Ethnicity			
Hispanic			
Yes	25	1.6	
No	2137	98.4	
Non-Hispanic White			
Yes	53	2.4	
No	2796	97.6	
Non-Hispanic Black			
Yes	12	0.7	
No	1727	99.3	
Other-Including Multi-Racial			
Yes	19	4.3	0.0014
No	492	95.7	

^1^ HS: high school diploma; ^2^ GED: General Educational Development test.
